# Numerical and Experimental Analysis of Stress–Strain Characteristics in DP 600 and TRIP 400/700 Steel Sheets

**DOI:** 10.3390/ma17010210

**Published:** 2023-12-30

**Authors:** Emil Evin, Miroslav Tomáš, Stanislav Németh

**Affiliations:** 1 Department of Automotive Production, Faculty of Mechanical Engineering, Technical University of Košice, Mäsiarska 74, 040 01 Košice, Slovakia; miroslav.tomas@tuke.sk; 2 USSE Research and Development, U.S. Steel Košice s.r.o., Vstupný Areál U.S. Steel, 044 54 Košice, Slovakia; snemeth@sk.uss.com

**Keywords:** advanced high strength steel, energy absorption, three-point bending, experiment, simulation

## Abstract

The body constitutes the largest proportion of the total vehicle weight. Recently, increasing efforts have been made towards reducing its weight and improving its crashworthiness. By reducing its weight, fuel consumption will be reduced, and this will also translate into lower CO_2_ emissions. In terms of safety, vehicle body components use high strength steel which can absorb a substantial amount of impact energy. The present study pays attention to DP 600 and TRIP 400/700 stress–strain characteristics at quasi-static strain rates. The stress–strain characteristics of absorption capacity, stiffness, and deformation resistance force were investigated experimentally by tensile tests, three-point bending tests, and numerical simulations. The results indicate the potential for increasing the absorption capacity, stiffness, and deformation resistance force of the vehicle body’s deformable steel components. The present study verified the possibility of replacing physical testing with numerical simulation. A reasonably satisfactory agreement between the experimentally determined stress–strain characteristics and the numerical simulation was achieved, which can reduce the development time of deformable vehicle body components, reduce costs and optimize the selection of materials. The results extend the state of knowledge on the deformation characteristics of high-strength materials and contribute to the optimization of body components in terms of passive safety and weight.

## 1. Introduction

In the automotive industry, major efforts are made to develop coherent and balanced car concepts combining safety, emissions and affordability requirements. Such a combination of factors is required to be achievable through realistic, technologically and affordably viable production methods while avoiding an excessive burden on the environment. One of the factors that largely determines the success or failure of a given car concept is its safety. In terms of passive safety, a crucial role belongs to the car body, which consists of a front crumple zone, a safe zone for passengers (safety cage) and a rear crumple zone. In the event of a collision, the deformation structure of the vehicle body’s front and rear sections is designed to absorb as much kinetic energy as possible so that the permissible biomechanical limit of overloading the human body is not exceeded in the event of a collision. The deformation of the structure at the front and rear of the vehicle is controlled. The forces generated by the impact in the bumper cross member are distributed to the longitudinal beams. From the longitudinal beams, the stress is transferred to the tunnel, to the sills, to the floor, to the door structure, and through the door pillar to the roof structure beams. In doing so, the longitudinal beams are subjected to a compressive force and the transverse beams and stiffeners are subjected to a bending force. The consequences of a side impact accident are often more severe than those of a frontal impact because the deformation structure of this part of the vehicle has to absorb the impact energy over a shorter path than in a frontal impact [1,2,3,4,5].

The absorption capacity (*EA*) of a deformable structure depends on the material used (*M*), the geometry of the deformable zone components (*G*) and the nature of the deformation force (*F_D_*). Different materials (steel, aluminum alloys, magnesium alloys and composite materials) are used in the body’s construction. The impact energy dissipation mechanisms of metallic and composite structures are significantly different. Structural parts of composite materials are brittle and dissipate energy through various combined fracture mechanisms (fiber breakage, matrix cracking, etc.). In contrast, metal structures allow energy dissipation by controlled elastoplastic deformation of the deformation structure components [6,7,8,9].

As mentioned above, the car body consists of a front crumple zone, a passenger safe zone and a rear crumple zone. Due to their required functionality, the material properties of the components used for each zone also differ. For the front and rear crumple zones, dual and multiphase steels (dual-phase steels—DP, complex-phase steels—CP, transformation induced plasticity steels—TRIP, twinning induced plasticity steels—TWIP) are used, which are equipped with very good *EA*. For the safe zone (safety cage), martensitic steels—MS, hot formed steels—HF, and dual-phase steels >1000 MPa are used, which are characterized by high strength [1,8,9,10,11].

Dual-phase steels (DP) consist of a fine-grained ferritic matrix in which martensite islands are dispersed. Combining these phases gives the material good fatigue resistance and toughness while maintaining good formability and weldability. DP steels are produced by controlled cooling from the austenitic phase or a two-phase ferritic and austenitic phase. A certain volume fraction of austenite is converted to ferrite, and on sudden cooling, the residual austenite is converted to martensite. The total martensite content in DP steels is in the range of 5–30%. As the proportion of the martensitic phase increases, the strength of the steel increases. High strain hardening exponent n values and high *EA* of impact energy characterize DP steels [8,12,13]. These steels are produced in standardized grades DP350/600, DP 500/800, and DP 600/980. They are used for floor parts, rear bulkheads and deformation stiffeners, front chassis frame, engine mounts, etc. [12].

High-strength multiphase steels with transformation-induced plasticity (TRIP) exhibit an excellent combination of strength and ductility. Their microstructure consists of islands of residual austenite (7–15%) and bainite (30–35%) dispersed in a soft ferritic matrix (50–55%). In some types of TRIP steels, martensite (1–5%) is present. During plastic deformation, austenite is transformed into martensite (TRIP effect). This effect is not concentrated in a particular area but is uniformly distributed throughout the volume. Plates of martensite formed in the slip planes are an obstacle to the movement of dislocations. A martensitic transformation is indicated at its front if a crack develops in the deformed zone during stressing. Plates of martensite then prevent further crack propagation [8,12,13,14,15,16,17]. TRIP steels are produced in the following standardized grades: TRIP 350/600, TRIP 400/700, TRIP 450/800 and TRIP 600/980. Due to their high *EA*, these steels are used for vehicle body deformable components (transverse and longitudinal beams, B-pillar stiffeners, bumpers, door sills, etc.) [12].

**Figure 1:**
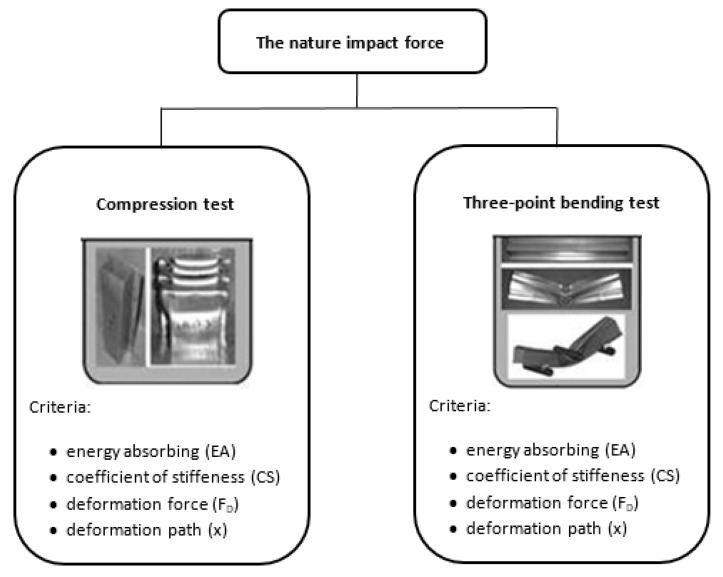
Scheme of the three-point bending test and compression test.

Designers and material engineers frequently face the dilemma of balancing the conflicting demands of vehicle design, weight and crashworthiness. When selecting an appropriate combination of materials for the body deformation structure components, designers need information on their resistance to deformation under compressive or bending forces (Figure 1) and information on their processability (formability, weldability, etc.)—Figure 1 [4,18,19,20]. 

This information can be predicted based on the results of the tensile test, three-point bending test, compression test (by squeezing the specimen) and numerical simulation. The present paper aims to draw attention to the possibilities of experimental and numerical methods to predict the stress–strain characteristics of materials such as deformation resistance force—*F_R_* or *F_D_*, the absorbed energy—*EA*, the nominal value of deformation resistance force—*F_MR_*, the efficiency of deformation resistance force—*E_FR_* and the characteristic of the stiffness of the deformation structure—*CS*. The above characteristics were investigated in the research conducted by tensile test, three-point bending test and numerical simulation of three-point bending [4,21,22,23].

## 2. Materials and Methods

### 2.1. Materials

**Figure 2:**
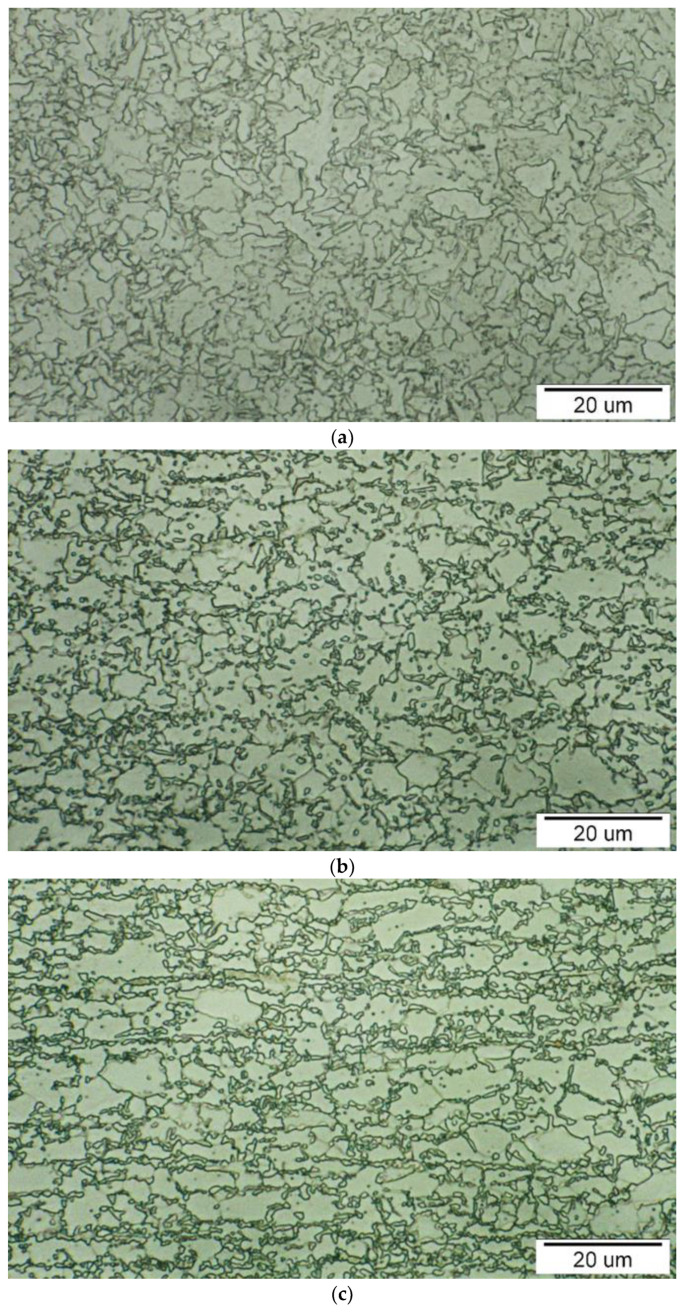
Microstructure of the investigated materials: (**a**) HSLA 240; (**b**) DP600; (**c**) TRIP 400/700.

The experimental research of stress–strain characteristics was conducted on sheets of micro-alloyed steel HSLA 240, dual-phase steel DP 600 and multiphase steel TRIP 400/700, which are used in the construction of car bodies. HSLA 240 micro-alloyed steel microstructure is formed by fine equiaxial ferrite grains and small pearlite and tertiary cementite formations—Figure 2a. The microstructure of DP 600 dual-phase steel is a fine-grained polyhedral formed by equiaxial grains of ferrite and very fine grains of martensite—Figure 2b. The TRIP 400/700 microstructure is fine-grained, ferritic, and homogeneous, in which the structural components are arranged in rows. Minority very fine formations of residual austenite and martensite occur in the structure—Figure 2c.

**Figure 3:**
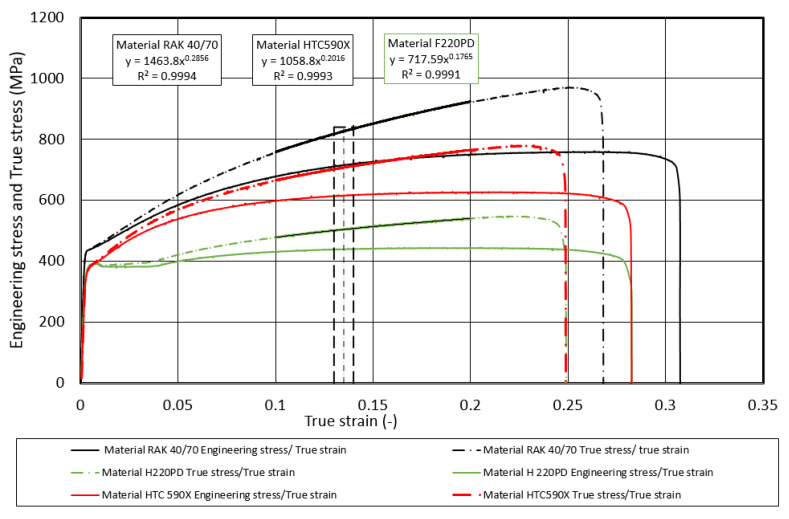
Dependence of engineering *σ_E_* and true *σ_T_* stress on true *φ_T_* strain.

The mechanical properties of the materials were determined by tensile testing on a TIRATEST 2300 testing machine (TIRA Maschinenbau GmbH, Rauenstein, Germany) under the provisions of ISO 6892-1:2019, ISO 10113:2020, and ISO 10275:2020 [24,25,26]. The TIRATES 2300 tensile machine is equipped with a tensometric load cell and extensometer. Five samples were tested in the experiment when the strain rate was 0.002 s^−1^. Then, average values of mechanical properties and their standard deviation were calculated from five measurements. Figure 3 shows the dependences of both the engineering stress *σ_E_* and the true stress *σ_T_* on the engineering strain *ε_E_* and the true strain *φ_T_*, which were obtained from the testing machine recordings. The measured values of the mechanical properties: yield strength *R_e_*, tensile strength *R_m_*, uniform ductility *A_U_*, total ductility *A_80_*, material constant *K_0.1–0.2_* and *n_0.1–0.2_* are presented in Table 1.

**Figure 4:**
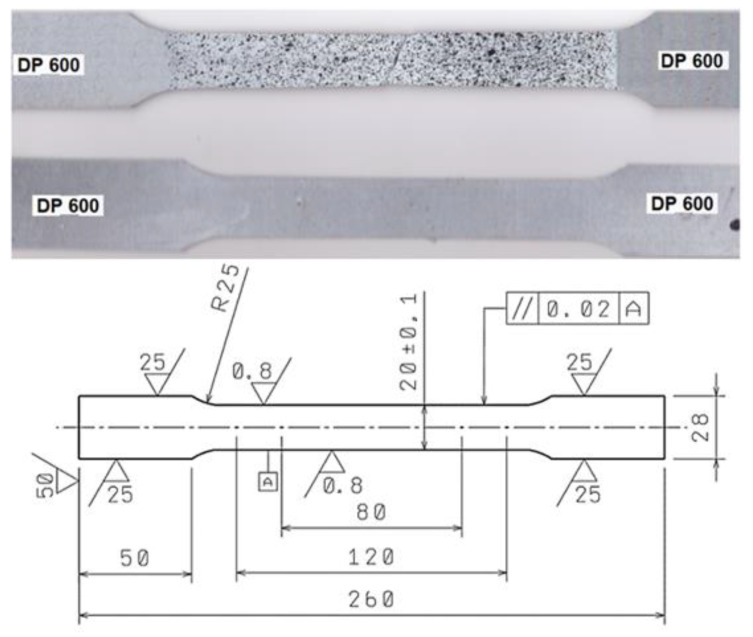
The sample before and after the tensile test when recorded by ARAMIS SRX optical system.

As the basic deformation characteristic of materials used in body construction, *EA* expresses the ability to absorb kinetic energy E_K_ into their failure. As it follows from the physio-metallurgical nature of strength and plasticity, *EA* is closely related to the material’s toughness [27,28]. Toughness can be characterized as the mechanical work related to the unit volume to failure of the material—*EA/V_0_*. *EA_E.t_/V_0_* can be determined from the tensile test record from the area under the *σ_E_* to *ε_E_* stress–strain curve—Figure 4, where the dependence of *σ_E_* on *ε_E_* is presented by the solid line and the dependence of *σ_T_* on *φ_T_* is presented by the dashed line. Then, *EA_Eng.t_/V_0_* was evaluated as follows:

(1) $$\frac{{EA}_{Eng.t}}{V_{0}}=\sum_{i=1}^{n}\left(\bar{\sigma_{E}\left(\varepsilon_{i}\right)}\right).∆\varepsilon_{E}$$

Similarly, the *EA_CTM.t_/V_0_* characteristics can be calculated by trapezoid method from the dependence of *σ_T_* on *φ_T_* as follows:

(2) $$\frac{{EA}_{CTM,t}}{V_{0}}=\sum_{i=1}^{n}\left(\bar{\sigma_{T}\left(\varphi_{i}\right)}\right).∆\varphi_{T}$$

As can be observed in Figure 3, the *σ_T_* to *φ_T_* curve is less flattened than the *σ_E_* to *ε_E_* dependence curve. This is probably since in the uniform strain area, the cross-section shrinks on one side, but on the other side, there is an intense strain hardening. At the point when σ_T_ starts to decrease, the formation of a neck and, consequently, the failure of the specimen occurs. The strain course in the area of neck formation was imaged with the ARAMIS 3-D optical system. The ARAMIS SRX measuring system with camera resolution of 12 Mpix and 8 GB of the internal memory was used for DIC testing. The maximum sensing speed was 335 Hz a few seconds before specimen fracture, while from the beginning the sensing speed was set to 20 Hz. The sample before the test without the speckles is shown on the bottom, and the sample with speckles on the white background after the fracture is shown on the top—Figure 4.

**Figure 5:**
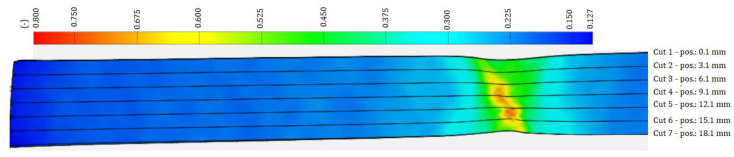
Deformation record by the ARAMIS system along the HSLA 240 specimen during the tensile test.

**Figure 6:**
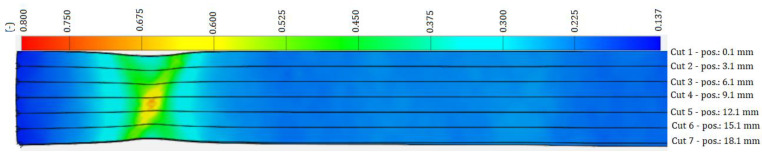
Deformation record by the ARAMIS system along the DP 600 specimen during the tensile test.

**Figure 7:**
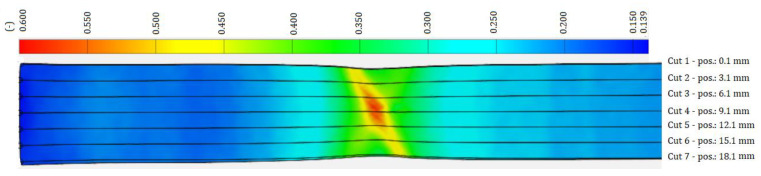
Deformation record by the ARAMIS system along the TRIP 400/700 specimen during the tensile test.

Figure 5, Figure 6 and Figure 7 shows a color map of the strain distribution at the point of deformation before failure of the sample. The strong (deep) blue color shows the undeformed area of the sample, the lighter blue color shows the area in which the localization of plastic deformation has not yet occurred, the green color shows the area of the beginning of the localization of plastic deformation, i.e., local narrowing of the cross-section of the sample begins (forming a neck), the yellow color shows the area of the formation of a gradual local narrowing of the cross-section, and the red shows the area just before the failure of the sample. The amount of plastic strain is expressed by the strains *φ_T1_*, *φ_T2_* and *φ_T3_* at the limit points SP, WP, CP—Table 2. As can be seen in Figure 5, Figure 6 and Figure 7, in the blue region, the width of the specimen is the same along the length of the specimen. In the blue region, it can be seen that there is a slight narrowing of the sample width in the deformation localization area, in the yellow region the narrowing of the width is more pronounced than in the green region, and in the red region in the middle of the sample the narrowing is the most pronounced. The strain development at the location of plastic deformation was expressed by the coefficient of normal anisotropy by the Lankford’s anisotropy parameter r, which is given by the ratio of the deformation in the direction of the width *φ_2_* of the sample to the deformation in the direction of the thickness *φ_3_* of the sample:

(3) $$r=\frac{\varphi_{2}}{\varphi_{3}}$$

The *r* values calculated for individual areas are shown in Table 2.

The deformation work *W_D_* respectively *EA_TC.t_* can be calculated as the area under the dependence curve *σ_T_* − *φ_T_* from the Equation (4):

(4) $$W_{D}={EA}_{CT.t}=S_{0}.L_{0}\int_{\varphi =0.002}^{\varphi_{i}}\sigma_{Ti}.d\varphi_{i}$$

If *σ_T_* from Krupkovsky Equation (5) is expressed as follows:

(5) $$\sigma_{Ti}=K.{\left(\varphi_{0}+\varphi_{i}\right)}^{n}$$

Consequently, after substituting *σ_T_* from Equation (5) into Equation (4), the following is the result:

(6) $$\frac{{EA}_{CT,t}}{V_{0}}=\int_{0.002}^{\varphi_{iF}}K.\varphi_{Ti,F}^{n}d\varphi =K.\frac{\varphi_{T,F}^{n+1}-0.002^{n+1}}{n+1}$$

From the measured values of forces and elongations, *σ_Ti_* values were calculated using Equation (7):

(7) $$\sigma_{Ti}=\frac{F_{Ti}}{S_{0}}.EXP\left(\varphi_{Ti}^{-1}\right)$$

Calculating *φ_Ti_* strains using the Equation (8):

(8) $$\varphi_{Ti}=ln\left(1+\frac{A_{U,i}}{100}\right)=ln\left(1+\frac{∆L}{L_{0}}\right)$$

where *F_Ti_* is a true instantaneous force; *φ_Ti_* is the true strain value (*φ_Ti_ = ln (1 + ∆L_i_/L_0_)*); *∆L_i_* is the elongation of the specimen under uniaxial tensile stress; *L_0_* is the specific length of the specimen before deformation; *S_0_* is the initial specimen cross-section; and *A_U,i_* is uniform ductility.

The values of *K, n* obtained by regression analysis at different intervals and the calculated values of *EA_TRA.t_/V_0_* using regression analysis under uniaxial tensile stress are given in Table 3.

**Figure 8:**
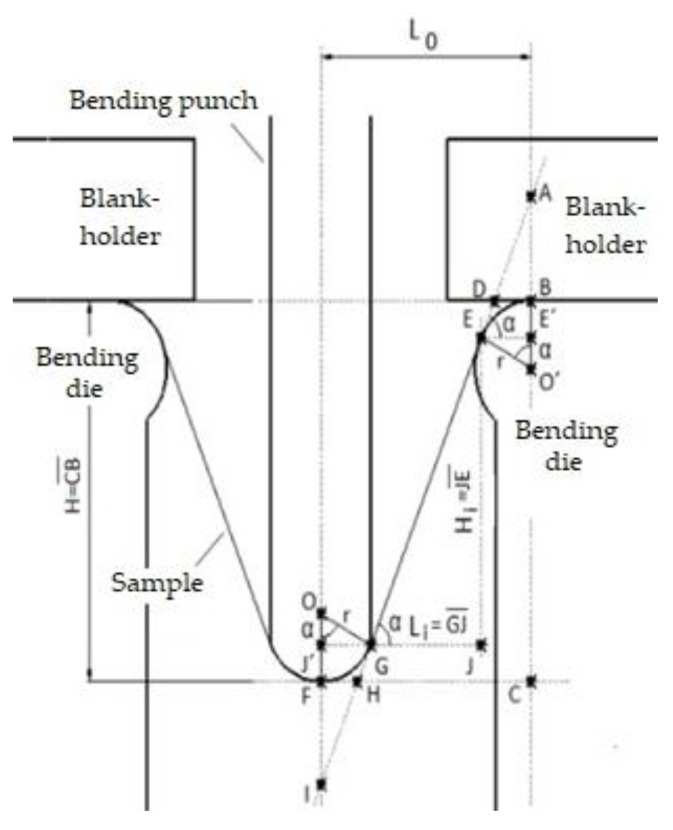
Principle scheme of the three-point bending test.

**Figure 9:**
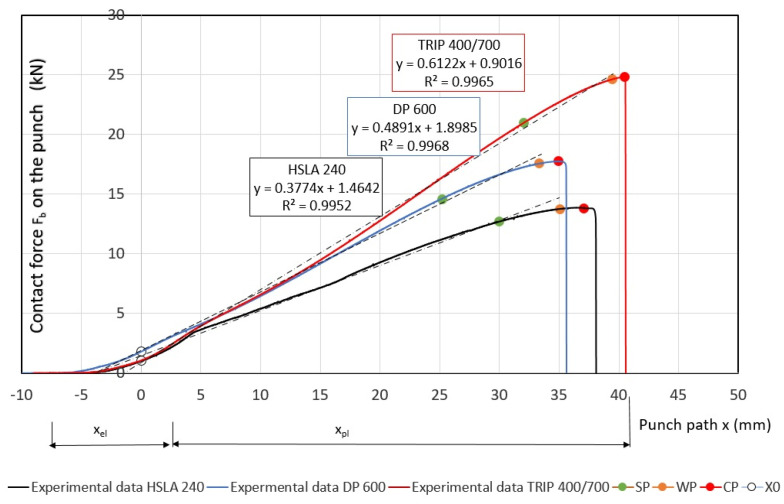
Record of the bending force on the punch path.

As mentioned above, in car collisions, the bumper, cross members, sill, B-pillar of the door and roof reinforcement are subjected to bending force. These parts are connected to the body frame structure. In order to make the bending stresses on the deformation components as close as possible to the actual stresses, the ends of the specimens were mechanically clamped in the jaws—Figure 8. For the experimental research, specimens with a width of 30 mm and a length of L = 300 mm were used. Before the deformation of the specimens, a deformation network was etched on their surface. The specimens were deformed to failure at a testing machine traverse speed of 10 mm.min^−1^. The bending punch and strain gauge sensor were attached to the moving crossbar of the TIRATEST 2300 testing machine. The machine allows recording the bending force *F_b_* and the traverse distance *x*—Figure 9. Points S0, SZ, WZ and CZ were marked on the curve of the dependence of *F_b_* on the strain path *x*, which delimit the individual deformable zones. *F_b_* in the range between points X0 and SZ increases linearly as a function of *x_pl_*. The area between these points can be referred to as the safe zone. This zone has a sufficient margin of plastic properties until failure. The curve starts to flatten gradually in the area between the SZ and WZ points, and a neck forms. This area can be described as a warning zone. In the area between the WZ and CP points, the curve of the dependence of *F_p_* on *x_pl_* is quite flattened. The reserve of plastic properties is exhausted. This area can be referred to as the critical zone. In the area beyond the CP point, the specimen is broken—fracture. The deformation work *W_D,b_* or the absorbed energy *EA_b.CTM_* was evaluated from the *F_b_* record of the *x_pl_* test by three-point bending combined stretching the specimen using Equation (9)—Figure 9:

(9) $$W_{D.b.CTM}={EA}_{b.CTM}=\int_{0}^{x_{max}}F_{b}\left(x_{pi}\right)dx\approx \sum_{i=1}^{n}\left(\bar{F_{b}(x_{i}}\right)∆x_{pl}$$

whereas $\bar{F_{b}\left(x_{i}\right)}$ is the average value of the force in the selected interval, $\bar{x_{i}}$ is the midpoint of the chosen interval [*x_i−1_,x_i_*], and Δ*x = x_i_ − x_i−*1*_*.

**Figure 10:**
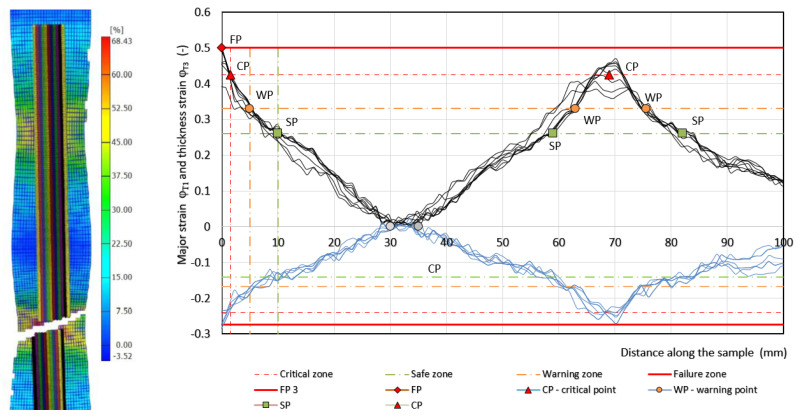
Record from the strain evaluation by the 3D optical system Argus—material HSLA 240. Note: The measured strain φ_1_ values are indicated by a thin solid black line and φ_3_ value are indicated by a thin solid blue line.

The vehicle body consists of a series of deformable zones [29], each designed to resist deformation until a certain stress level on the part is reached. The deformation elements’ resistance force (*F_R.b_ = F_b_*) to plastic deformation is limited by the material properties and geometry. A larger resistance (*F_R.b_*) of a deformation element under impact increases the deceleration, which may cause the element to exceed its biomechanical limits. It can be seen from Figure 10 that the area under the curve of the dependence of the bending force Fb on the maximum deformation path *x_pl_*. max can be simply calculated as the area of a triangle as follows:

(10) $${EA}_{b.F}=\frac{F_{bmax.}x_{plma}}{2}$$

A *F_bmax_* can be expressed as a function of the stiffness characteristic:

(11) $$F_{bmax}={CS}_{b.F}.x_{plmax}$$

If we substitute Expression (11) for *F_bmax_* in Equation (10), we derive the following Equation (12):

(12) $${EA}_{b.F}=\frac{1}{2}{CS}_{b,F}.x_{pl}^{2}$$

It is possible to determine the stiffness characteristic more precisely if we use *EA_b.CTM_* for *EA_b_._F_*. Then, from Equation (12), after adjustment, we arrive at Equation (13):

(13) $${CS}_{b.EA}=\frac{2.{EA}_{b.CTM}}{x_{pl}^{2}}$$

Regression analysis can also determine the stiffness characteristics of *CS_b,RA_*. When applying it, the following principles should be observed [30]:

- the interval of the strain path x_pl_ in linear regression should be chosen as wide as possible,
- the R^2^ value of the variance in regression analysis should be greater than >0.95.

Using the stiffness characteristic *CS_b,RA_* determined by regression analysis, Equation (14) can be used to calculate *EA_b,RA_/V_0_* as follows:

(14) $${EA}_{b.RA}=\frac{{CS}_{b,RA}}{2}.x_{pl}^{2}$$

The determination of the stiffness characteristics *CS_b.RA_* for each material by regression analysis is shown in Figure 9. The measured values of *F_b_ = F_R.b_, x_pl_*, calculated values of stiffness characteristics *CS_b.RA_, CS_b.EA_* and *EA_b.CTM_* are presented in Table 4.

**Figure 11:**
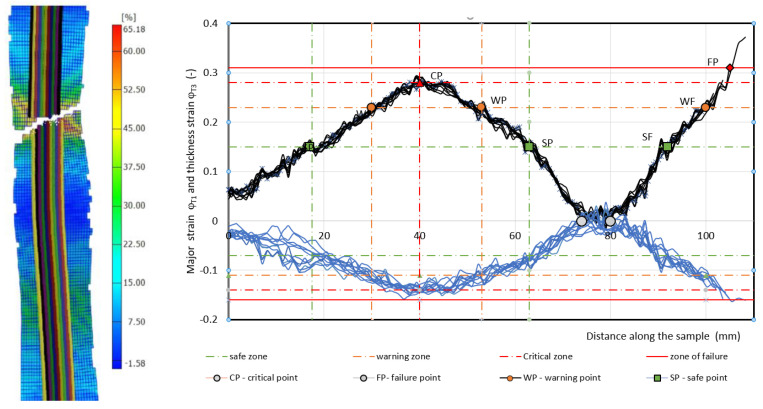
Record from the strain evaluation by the 3D optical system Argus—material DP 600. Note: The measured strain φ_1_ values are indicated by a thin solid black line and φ_3_ value are indicated by a thin solid blue line.

**Figure 12:**
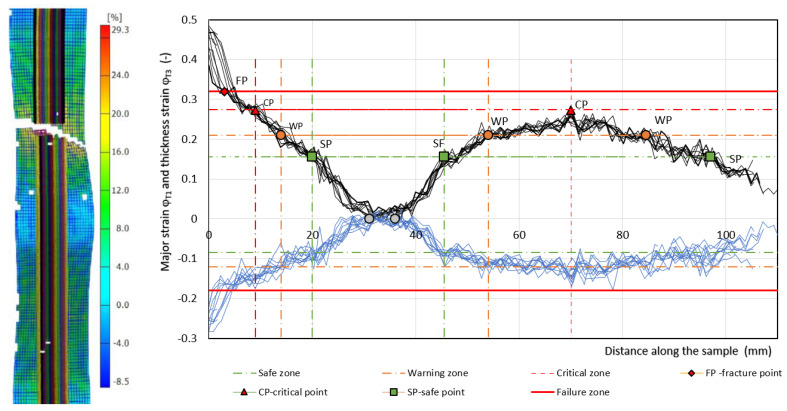
Record from the strain evaluation by the 3D optical system Argus—material TRIP 400/700. Note: The measured strain φ_1_ values are indicated by a thin solid black line and φ_3_ value are indicated by a thin solid blue line.

The failure of the specimens occurred on one side in the center of the unsupported area of the specimen, and on the other side of the unsupported specimen, there was only a narrowing of the specimen cross-section—Figure 10, Figure 11 and Figure 12. From the deformation grid etched on the specimen, the in-plane φ_T1_ and φ_T2_ along the specimen in 11–12 sections were evaluated by the Argus 3D optical system as it is shown in Figure 10, Figure 11 and Figure 12.

The value of the third true strain *φ_T3_*, i.e., the thickness strain of the specimen, was calculated based on the validity of the volume constancy law according to the Equation (15):

(15) $$\varphi_{T1}+\varphi_{T2}+\varphi_{T3}=0$$

The Equation (15) after adjusting *φ_T3_*:

(16) $$\varphi_{T3}=-(\varphi_{T1}+\varphi_{T2})$$

The measured strain values at the boundaries of each zone are shown in Table 5.

### 2.2. Numerical Simulation of the Three-Point Bending Test

**Figure 13:**
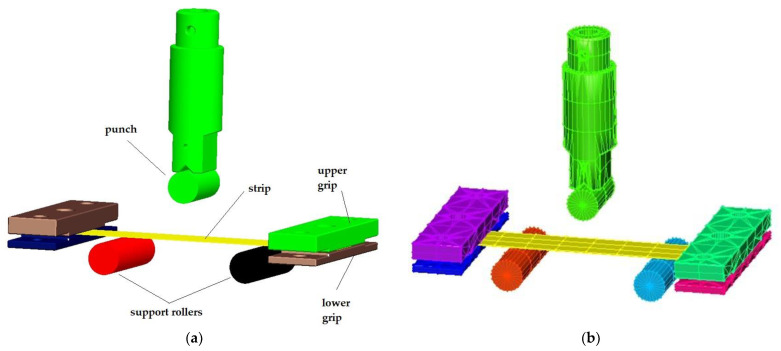
Three-point bending test with fixed ends: (**a**) 3-D model created in Creo 2.0; (**b**) simulation model created in Pam Stamp software.

To predict the stress–strain characteristics of the materials used, a numerical simulation of the bending test with fixed ends was also used in the PAM STAMP 2G V2012 software environment. A 3D model of the actual physical bending tool created in Creo 2.0 was used to set up the numerical simulation—Figure 13a. This was saved in *igs format, and the individual functional parts of the tool were imported into the simulation software. A specimen (strip of sheet metal) with dimensions corresponding to the real specimen (with a length of 120 mm, a width of 30 mm and a thickness of 0.75 mm) was also modelled. After importing them into the PAM STAMP 2G V2012 environment, the different parts of the tool were set to a position that corresponded to the real process. Subsequently, the properties such as rigidity, movement, friction, etc. of individual parts of the tool were specified—Figure 13b.

The distribution of stresses and strains depends on the behavior of the material. The authors [31,32] describe three models of the elastic–plastic behavior of the material in bending of the cross member: elastic–perfect plastic, elastic–linear plastic, and power-law hardening. The material properties in our numerical simulations were defined by the Hill-48 plasticity condition in combination with the Krupkovsky isotropic hardening model (Equation (5)). The values of Young’s modulus of elasticity *E*, Poisson’s number *µ*, strength constant *K*, strain hardening exponent *n* and the plastic strain ratios *r* were specified in the material card, and the boundary conditions (friction coefficient, loading direction, holding force, punch speed, etc.) were set in the process tree of the simulation software [33]. Surface blank was meshed with square elements of 0.5 mm size and the mesh was checked with no errors.

**Figure 14:**
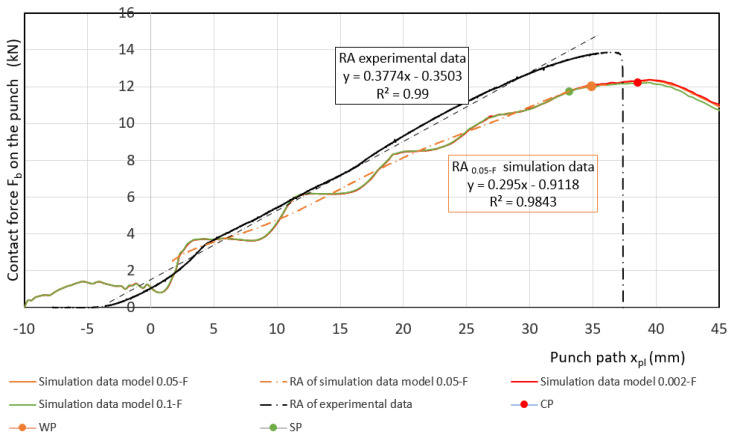
Comparison of simulation and experiment results for material HSLA 240.

**Figure 15:**
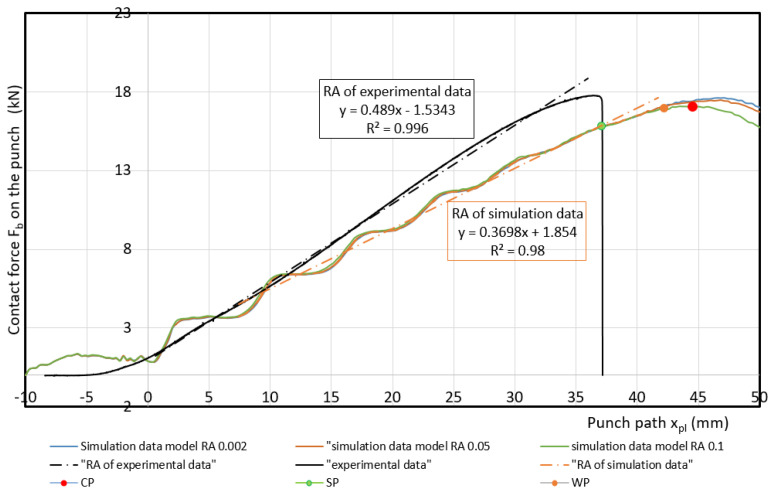
Comparison of simulation and experiment results for material DP 600.

**Figure 16:**
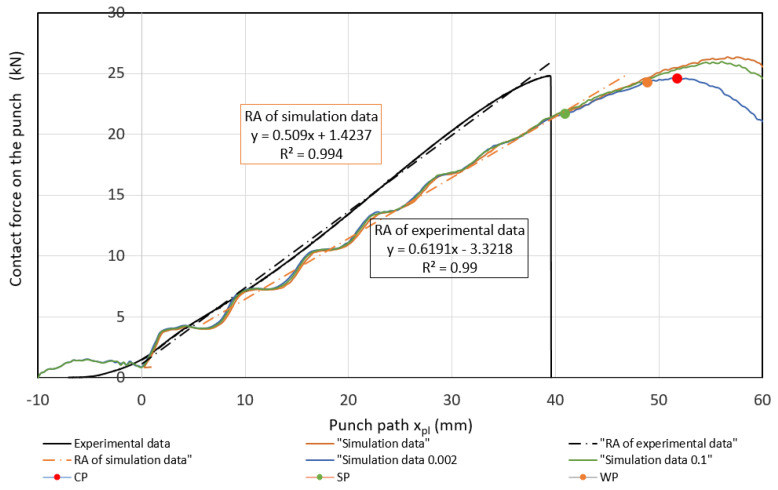
Comparison of simulation and experiment results for material TRIP 400/700.

Measured values of materials—strength constant *K*, strain hardening exponent *n* and Lankford’s anisotropy parameter *r* (Table 4)—were set as input values in the numerical simulation of three-point bending. Subsequently, the boundary conditions corresponding to the conditions of the three-point bending test were set. Before the true strain, the plate strip (specimen) was held at the edge by a force (300 kN) to prevent it from pulling out of the jaw. Subsequently, a bending path of 120 mm was defined. The deformation of the specimen was followed by the actual calculation of the stress–strain characteristics (*F_b,xpl_*, in-plane strains *φ_1T_, φ_2T_* and the thickness strain *φ_3T_*) in 11 steps. Figure 14, Figure 15 and Figure 16 show the dependences of the bending force *F_s.b_* on the punch path *x_pl.s_*, which were obtained by numerical simulation. The elastic *x_el_* and plastic *x_pl_* phases of the strain path are indicated in these figures. When evaluating *EA_S.b_*, the stress–strain characteristics were shifted to the left by *x_el_* (approximately 8 to 10 mm) i.e., by the restitution work (elastic work *EA_el_*, etc.)—Figure 14, Figure 15 and Figure 16. 

Similar to the above, the deformable zones were marked on the curve of the dependence of the deformation force *F_b_* on the strain path *x_pl_*. The dependence of *F_s.b_* on *x_pl.s_* is linear in the area between the CP and SP points. This zone can be referred to as the safe zone. In this zone, it is possible to fully exploit the properties of metallic materials in controlling the deformation process of body parts in impact. In the area between the SP and WP points, the dependence of *F_s.b_* on *x_pl.s_* begins to flatten and the plastic deformation gradually localizes. The specimen at the point of localization begins to narrow. This zone can be referred to as the warning zone (i.e., edge zone), in which the plastic deformation process of the deformed body parts can also still be controlled. In the critical zone between the WP and CP points, a more pronounced flattening of the *F_s.b_* vs, *x_pl.s_* dependence occurs. At the CP point, the plastic properties are exhausted; hence, the possibility of controlling the deformation process of body parts during impact is lost. Table 6 shows the measured values of *x_pl.s_*, *F_s.b_*, *EA_s.b_*, *CS_s.b_* in the plastic deformation area. In evaluating these characteristics, the same procedure was used to evaluate the experimentally determined stress–strain characteristics of the materials used.

## 3. Results and Discussion

**Figure 17:**
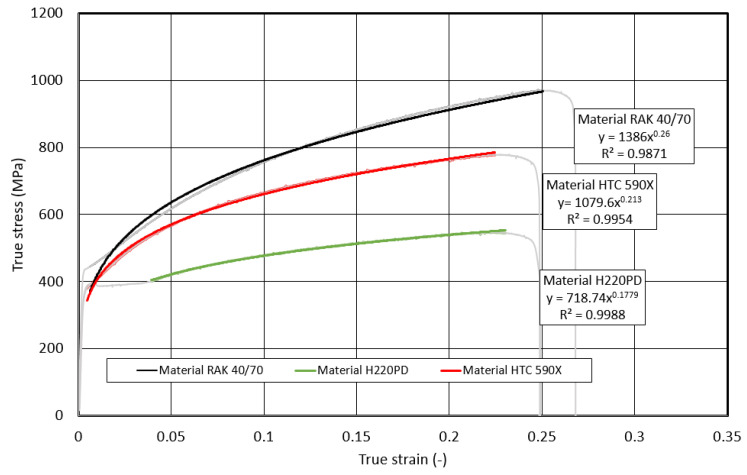
Dependence of the true stress on the true strain within the strain interval <0.002; φ_T,F_>.

**Figure 18:**
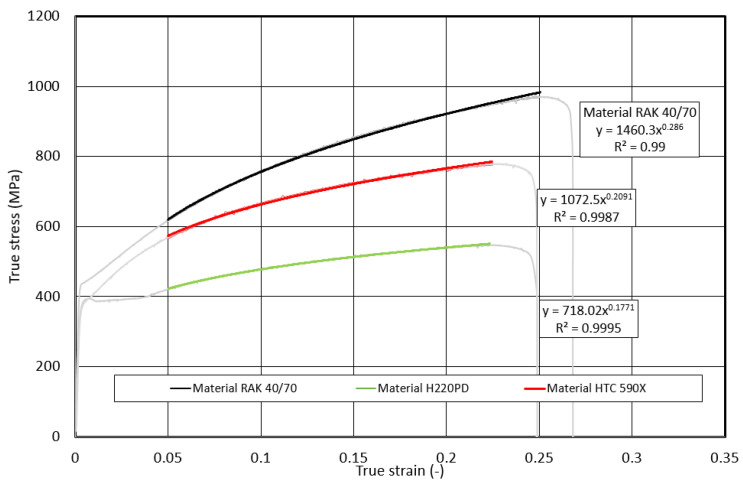
Dependence of the true stress on the true strain within the strain interval <0.05; φ_T,F_>.

**Figure 19:**
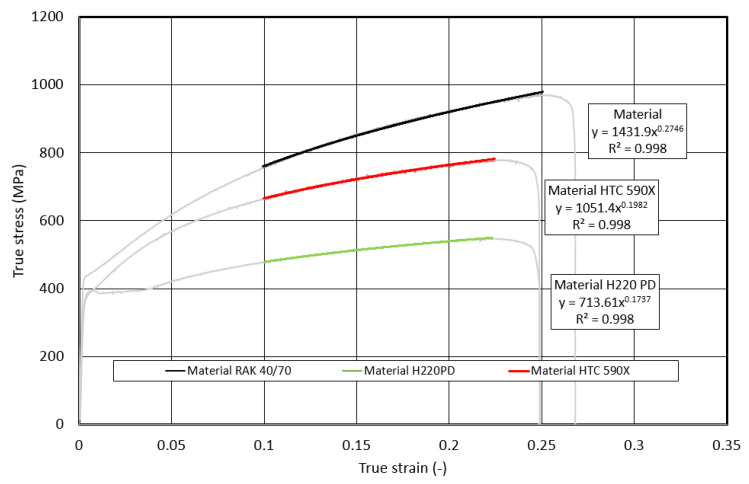
Dependence of the true stress on the true strain within the strain interval <0.1; φ_T,F_>.

The basic deformation characteristic of the materials used in the construction of the body is the ability to absorb (*EA*) kinetic energy (*EK*) upon impact to material failure. As follows from Equation (1), the lower predictive ability of *EA_Eng.t_* is related to the fact that the initial *S_0_* cross-section and not the actual *S_T_* cross-section is used in its determination. The predictive ability of the dependence *σ_T_* on *φ_T_* using the Krupkovsky equation was also verified outside the interval recommended by ISO 10275:2020 [26], namely in the following strain intervals: *φ_T_* from 0.002 to the uniform elongation *A_U_* (pre-break *φ_T,F_*), *φ_T,F_* from 0.5 to the uniform elongation *A_U_* (pre-break *φ_T,F_*), and *φ_T_* from 0.1 to the uniform elongation *A_U_* (fracture or pre-break *φ_T,F_*)—Figure 17, Figure 18 and Figure 19. 

**Figure 20:**
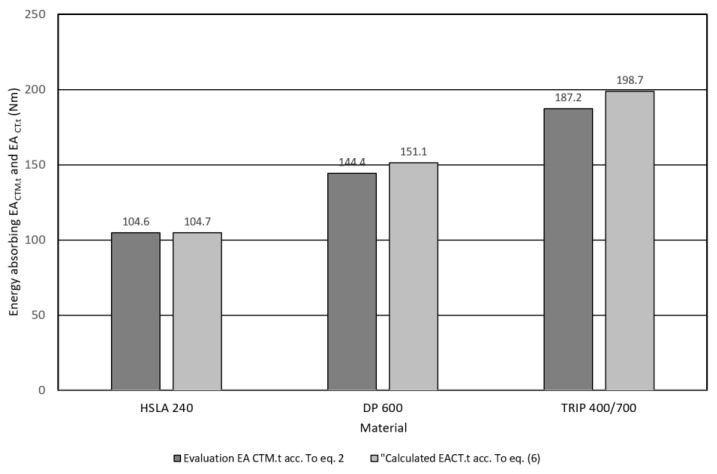
Comparison of the absorption energy of the investigated materials on the basis of applied models—tensile test.

The designer’s goal during the process of car body design is to reduce its weight and increase resistance to impact. Criteria for optimization for the impact resistance of body components are energy absorption, stiffness, etc. The absorption capacity *EA_CTM.t_* and *EA_b.CTM_* of the investigated materials was determined from the record of the tensile test and the three-point bending test using the trapezoidal method using Equations (2) and (9). The results obtained by the trapezoidal method were considered as a reference when comparing several assessment methods. The Figure 20 shows a comparison of the absorption capacity of micro-alloyed steel HSLA 240, two-phase steel DP 600, and multiphase steel TRIP 400/700 from the tensile test record.

**Figure 21:**
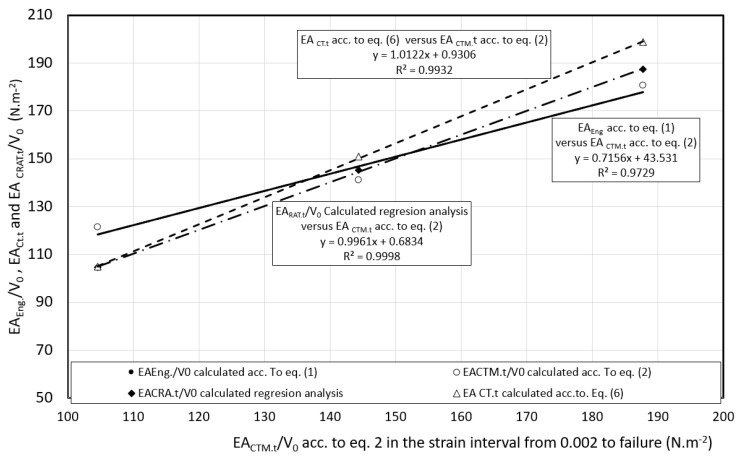
Dependence of the energy absorbing calculated from the regression model, according to Equation (6) models and according to Equation (1) on the energy absorbing according to Equation (2).

From the comparison of *EA_CTM.t_* DP 600 against micro-alloyed steel HSLA 240, it can be seen that this characteristic has increased by 30% and compared to TRIP 400/700 by up to 79%. The increase in absorption capacity of DP 600 and TRIP 400/700 steel compared to micro-alloyed steel is related to the strength coefficient *K* and strain hardening exponent *n*. In the case of DP 600, the value of *K* was greater by 47% and in the case of n by 13%. In the case of TRIP 400/700, the value of *K* was greater by up to 204% and *n* was greater by 61%. From the mentioned comparison, it follows that materials with a two- and multiphase structure strengthen more intensively than materials with a single-phase ferritic structure. The evaluation of *EA_CTM.t_* by the trapezoidal method is accurate but time-consuming, therefore the Relation (6) was derived. From the comparison of *EA_CTM.t_* and *EA_CT.t_* values calculated according to Relation (6) shows that agreement between these characteristics was achieved in the range from 1% to 6%. When qualitatively evaluating the dependence model *σ_T_* on *φ_T_* on Figure 21, we observe that *R*^2^ for the dependence of *EA_RA.t_/V_0_* and the measured values of *EA_CTM.t_/V_0_* takes the value of 0.9998. Similarly with the dependence of *EA_CT.t_/V_0_* on *EA_CTM.t_ R*^2^ was 0.9932. The dependence of *EA_CT.t_/V*_0_ on *EA_CTM.t_* shows a tendency that can be expressed as follows:

(17) $${EA}_{CT.t}/V_{0}=1.0212.{EA}_{CTMT.t}/V_{0}+0.931$$

If this tendency is confirmed or, if it could be, refined, then after substituting for *EA_CT.t_/V_0_* the expression from the right side of Equation (6) we derive:

(18) $$K.\frac{\varphi_{T,F}^{n+1}-0.002^{n+1}}{n+1}=1.0212.{EA}_{CTMT.t}/V_{0}+0.931$$

**Figure 22:**
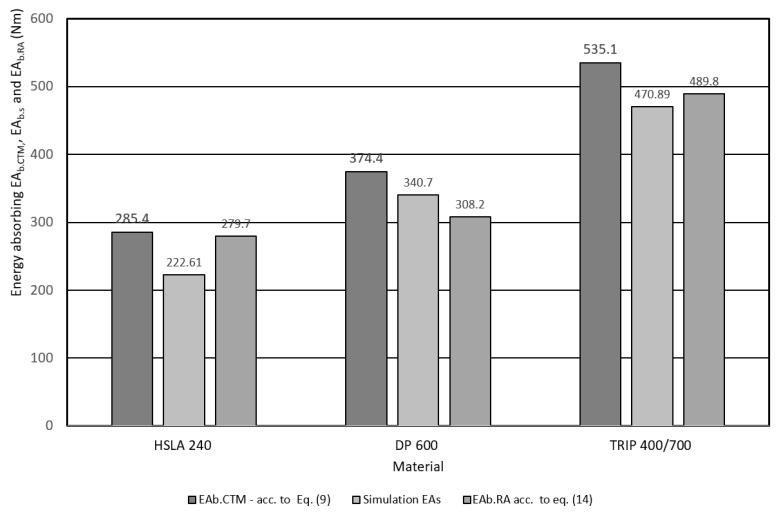
Comparison of the absorption energy of the investigated materials on the basis of applied models—three-point bending test.

Deformable parts of the body (bumper, B-pillar, door and roof reinforcements, etc.) are exposed to a bending force during an impact. In this case, the results of *EA_b.CTM_* evaluated by the trapezoidal method were considered as a reference when comparing. The absorption capacity (*EA_b.CTM_*) of the materials used was evaluated from the recording of the dependence of the bending force (*F_b_*) on the deformation path *x*. The Figure 22 shows a comparison of *EA_b.CTM_* of the investigated materials. From the comparison, it can be seen that the material DP 600 shows 1.3 times greater absorption potential and the material TRIP 400/700 approx. 1.9 times greater impact energy absorption potential than HSLA 240 material. 

**Figure 23:**
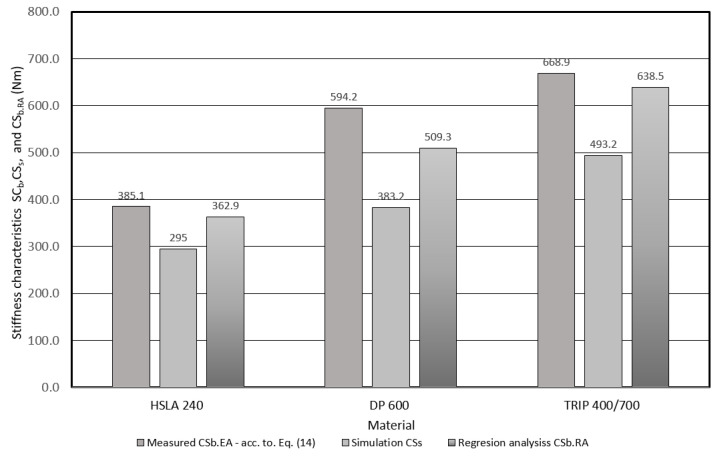
Comparison of the stiffness characteristics of the investigated materials based on the applied models—three-point bending test.

Another deformation characteristic is stiffness (*CS*). The *CS_b.EA_* value calculated according to Relation (13) was considered as a reference value when comparing the stiffness of the investigated materials. It can be seen from the *CS_b.EA_* comparison shown in the Figure 23, that DP 600 material exhibits 1.54 times greater stiffness and TRIP 400/700 approximately 1.74 times greater stiffness than HSLA 240. Based on the above, it can be assumed that DP 600 materials and TRIP 400/700 have the potential to reduce the weight of body parts (using thinner wall thickness components) while maintaining or even improving impact resistance properties compared to HSLA 240.

Furthermore, the possibility of using regression analysis in determining the characteristics of *CS_b.RA_* was verified. A comparison of *CS_b.RA_* and *CS_b.EA_* characteristics show that the agreement between these characteristics ranges from 86% to 95%. The possibility of predicting *EA* and *CS* was also verified based on the results of numerical simulation. For *EA*, concordance with *EA_s.b_* ranged from 86% to 90%, and for *CS_.s.b_* ranged from 64% to 67%. The issue of verifying the results of numerical simulation will be addressed in further research. 

The characteristics of *EA* and *CS* can guide designers when selecting materials for deformable body parts. For example, if *F_b_* loads the deformation element, the deceleration can be expressed from the energy balance condition:

(19) $$E_{K}=W_{D,b}={EA}_{b.CTM}$$

Assuming that a vehicle of weight m moves on a path *x_pl_* with acceleration a acquires kinetic energy:

(20) $$E_{K}=\int_{0}^{x_{pl}}m.a.dx=m.a.x_{pl}$$

This E_K_ on impact is consumed by the *W_D_* or absorbed by the deforming structure in the *x_pl_* path. The vehicle moves on this path with a deceleration a. When substituted into the energy equilibrium condition (Equation (19), we obtain:

(21) $$m.a.x_{pl}=\frac{{CS}_{b.EA}.x_{pl}^{2}}{2}$$

After adjusting the deceleration *a* is as follows:

(22) $$a=\frac{{CS}_{b.EA}.x_{pl}^{}}{2.m}$$

The effect of material substitution on deceleration can be expressed by the deceleration change index *Ia*:

(23) $$I_{a}=\frac{a_{i}}{a_{ref}}$$

whereas *a_i_* is a deceleration in the use of innovative material, *a_ref_* is the deceleration when using the reference material.

If we assume that on the same vehicle (of the same weight), the reference material of micro-alloyed HSLA 240 steel will be replaced with DP 600 dual-phase steel, then after substituting for a_i_ in Equation (23) and adjusting, the result is as follows:

(24) $$I_{a\;DP600}=\frac{{CS}_{b.EA\;DP600}}{{CS}_{b.EA\;HSLA240}}=\frac{0.441}{0.338}=1.30$$

When replacing DP 600 with TRIP 400/700 steel:

(25) $$I_{a\;TRIP400/700}=\frac{{CS}_{b.EA\;TRIP400/700}}{{CS}_{b.EA\;HSLA240}}=\frac{0.571}{0.338}=1.69$$

After substitution for *a_RA.b_*:

(26) $$I_{aRA,b\;DP600}=\frac{{CS}_{RA.b\;DP600}}{{CS}_{RA.b\;HSLA240}}=\frac{0.482}{0.361}=1.33$$

Similarly, when replacing DP 600 with TRIP 400/700 steel:

(27) $$I_{aRA,b\;TRIP400/700}=\frac{{CS}_{RA.b\;TRIP400/700}}{{CS}_{RA.b\;HSLA240}}=\frac{0.607}{0.361}=1.68$$

The comparison of the *I_a_* indices calculated using the *CS_b.EA_* Equation (13) and the regression analysis *CS_b.RA_* indicates that the differences are very small, ranging from −1% to 3%. When substituting HSLA 240 with DP 600, a change in the deceleration index of up to 30% can be expected, and when substituting HSLA 240 with TRIP 400/700, a change in deceleration of up to 69% can be expected.

Another important characteristic is the strain path *x_pl_*, at which controlled deformation of the deformation structure occurs. From the comparison of the experimentally measured *x_pl_* values and the calculated *x_pl,s_* shown in Table 6, it can be observed that there was a difference of 4% for the HSLA 240 material with a single-phase structure and a difference of 27% to 32% for the DP 600 material with a two-phase structure. The largest difference between *x_pl_* and *x_pl,s_* was observed for the TRIP 400/700 material with a three-phase structure (30% to 43%)—Table 6. We assume that these differences are related to the model used for prediction in the program file. Although plastic deformation is localized in the warning zone between the SP and WP points (the beginning of the neck formation was observed on the specimen), it is still possible to control the body parts’ deformation processes at impact effectively.

**Figure 24:**
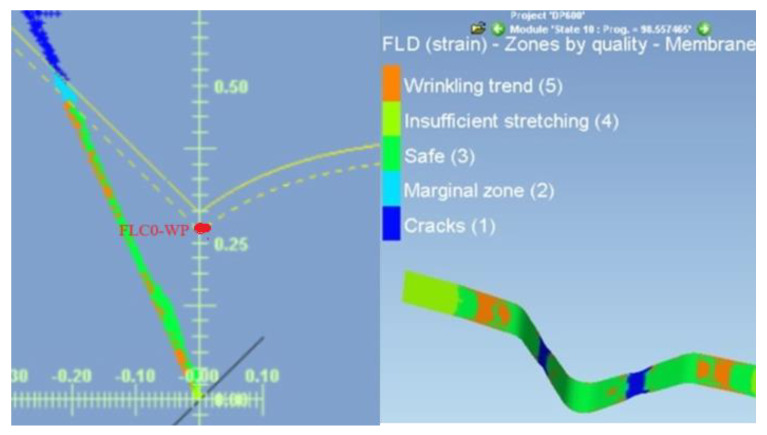
Determination of FLC0 by numerical simulation—material DP 600.

For predicting deformation states can be used FLC [34,35,36]. These curves allow the separation of the different transition zones from the safe deformation behavior of the material (with a certain margin) to the states where plastic deformation is localized, i.e., neck formation and subsequent failure. In FLC design, it is important to know the value of *FLC0* i.e., the state when at *φ_2T_* = 0 the value of *φ_1T_* (*FLC0 = φ_1T_*) is determined. The example is shown in Figure 24 for DP 600 material. Based on the results of [34], the value of *FLC0* for HSLA 260 material is about 0.3, based on [35], the value of FLC0 for DP 600 material is about 0.2 and based on [36] for TRIP 400/600 material the value is about 0.2. These *FLC0_CP_* values for separating the critical zone from the warning zone (FLC0_WP_) are recommended to be shifted by 10%—Table 7. 

The curve that separates the critical zone from the warning zone is indicated in the FLD by a dashed line—Figure 24. A comparison of the numerical simulation results presented in Table 7 with the experimental results published in [32,33,34] shows for the HSLA 240 material, with the assumed strain *φ_2WP_* = 0, the FLD corresponds to *FLC0_WP.s_* = 0. 27; for the DP 600 material the value *FLC0_WP.s_* = 0.18 [33]; and for the TRIP 400/700 material the value *FLC0_WP.s_* = 0.18 [34]. The above comparison shows that the differences of *FLC0_WP.s_* obtained by simulation for the DP 600 and TRIP 400/700 material are 50% as large as the data published in [35,36].

**Figure 25:**
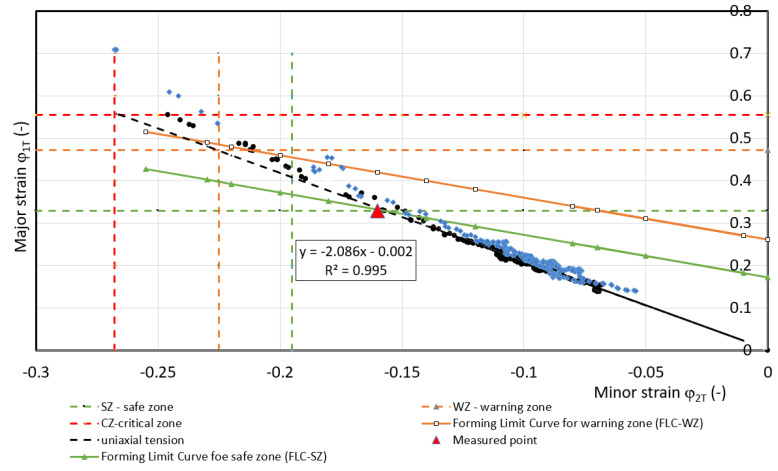
Dependence of *φ_1T_* on *φ_2T_* for material HSLA 240.

**Figure 26:**
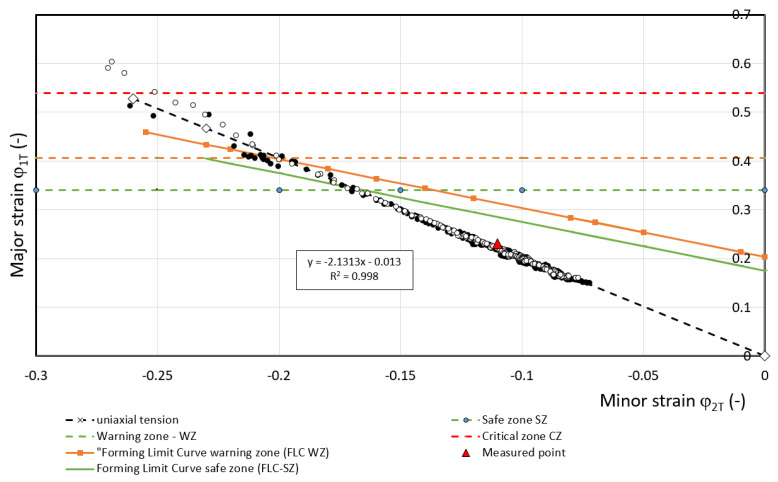
Dependence of *φ_1T_* on *φ_2T_* for material DP 600.

**Figure 27:**
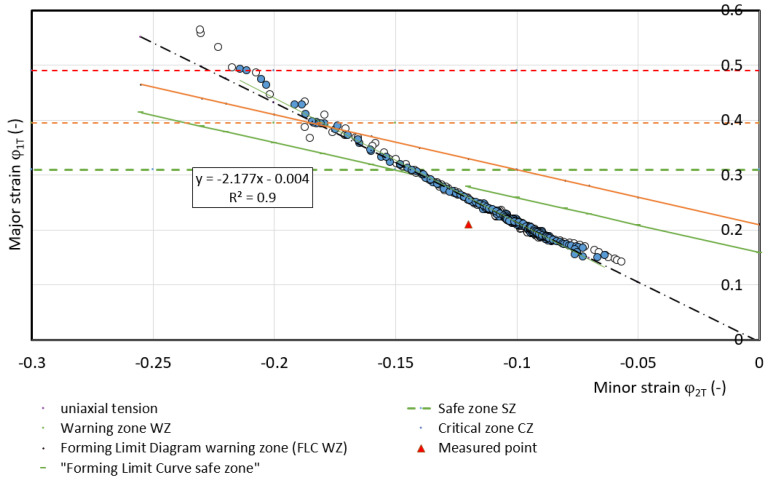
Dependence of *φ_1T_* on *φ_2T_* for material TRIP 400/700.

Based on the strain measurements *φ_1T_* and *φ_2T.b_* using ARAMIS on the tensile test specimens, the *FLC0_WP_* value for the warning zone was evaluated—Table 7. The WP point defined the strain at the interface between the warning zone. At point WP, the measured strain values *φ_1T_* and *φ_2T_* started to deviate from the directive of their interdependence (*R*^2^ > 0.99), or the difference of the values of the normal anisotropy coefficient *r* was greater than 0.01. This difference then increased more and more—Figure 25, Figure 26 and Figure 27. From the combination of the values *φ_1T_* and *φ_2T_*, the value of *FLC0_WP_* was calculated at the defined point WP from the well-known equation [37]:

(28) $$\varphi_{1Ti}={FLC0}_{WP}-\varphi_{2Ti}$$

Calculated values of *FLC0_WP_* are presented in Table 7.

## 4. Conclusions

The deformation properties were analyzed for HSLA 240, DP 600 and TRIP 400/700. From the results obtained, it was found that for the *EA* prediction of the materials used, it is appropriate to use the material constants *K* and *n* determined using the Krupkovsky regression model, which showed a 99.98% agreement between the measured *EA_CTM.t_* values and the calculated *EA_RA.t_* values. The results indicate that *EA_RA.t_*, *EA_b.RA_* and *CS_b.RA_* strongly correlate with the material constant *K* and *n*. Further, it can be concluded that DP 600 and TRIP 400/700 materials can potentially reduce intrusion into the passenger compartment. It should also be noted that when replacing (material upgrading) the micro-alloyed HSLA 240 steel with DP 600 or TRIP 400/700 dual-phase steel, the human body will be overloaded during impact. The results indicate that the *EA_RA.t_*, *EA_b.RA_* and *CS_b.RA_* characteristics depend on the microstructure state (single-phase, dual-phase or multiphase, phase transformations, etc.) occurring during deformation. Future research will focus on the effect of strain rate, the nature of the deformation force, the geometry of the deforming components and the application of composite materials on the stress–strain characteristics of metallic and non-metallic materials. From a numerical simulation point of view, the focus will be on material models and the detection of input data on material properties (*K*, *n*, FLC, friction, etc.).

The benefit of this article lies in the proposal methodology for predicting the impact characteristics of body parts loaded with bending force based on the mechanical properties of materials determined by tensile testing.

## Figures and Tables

**Table 1:** Measured values of mechanical properties of the materials used.

	Mechanical Properties							
	R_e_	R_m_	A_u_	A_80_	K_0.1–0.2_	n_0.1–0.2_	EA_CTM.t_/V_0_	EA_CT.t_/V_0_
Material	(MPa)	(MPa)	(%)	(%)	(MPa)	(-)	(N.mm) Acc. to Equation (2)	(N.mm) Acc. to Equation (6)
HSLA 240	388 ± 2	449 ± 1	17 ± 1	29 ± 1	719 ± 3	0.178 ± 0.002	104.6	104.7
DP 600	376 ± 4	632 ± 2	19 ± 0.5	28 ± 0.5	1059 ± 2	0.202 ± 0.001	144.4	151.1
TRIP 400/700	440 ± 3	764 ± 2	25 ± 1	30 ± 2	1468 ± 6	0.287 ± 0.002	187.2	198.7

Note: yield strength *R_e_*, tensile strength *R_m_*, uniform ductility *A_U_*, total ductility *A_80_*, *K_interval_*—strength coefficient, *n_interval_*—strain hardening exponent, *0.1–0.2*—interval 0.1 within to 0.2 strain, strength coefficient *K_0.1–0.2_*, *n_0.1–0.2_* strain hardening exponent, *EA_CTM.t_/V_0_* absorbed energy calculated by trapezoidal method according to Equation (2) from the tensile test record from the area under the *σ_T_* to *φ_T_* true stress–true strain curve, *EA_CT.t_/V_0_* absorbed energy calculated according to Equation (6) from the tensile test record from the area under the *σ_T_* to *φ_T_* true stress–true strain curve, *V_0_* unit volume.

**Table 2:** The strain values measured with the Aramis optical system.

Strain Zone	Strain	Material			Lankford’s Anisotropy Parameter r (-)		
		HSLA 240	DP 600	TRIP 400/700			
Safe point SP (green)	Major φ_T1_	0.365	0.370	0.310			
	Minor φ_T2_ (-)	−0.166	−0.184	−0.143	0.84	0.98	0.86
	Thickness φ_T3_ (-)	−0.299	−0.187	−0.167			
Warning point WP (yellow)	Major φ_T1_ (-)	0.540	0.404	0.380			
	Minor φ_T2_ (-)	−0.245	−0.200	−0.180	0.83	0.98	0.86
	Thickness φ_T3_ (-)	−0.295	−0.204	−0.210			
Critical point CP (red)	Major φ_T1_ (-)	0.640	0.635	0.490			
	Minor φ_T2_ (-)	−0.260	−0.275	−0.210	0.68	0.86	0.75
	Thickness φ_T3_ (-)	−0.380	−0.360	−0.280			

**Table 3:** Measured values of n, K and energy absorbing at different intervals of true strain.

Material	K_0.002–F_ (MPa)	n_0.002–F_ (-)	K_0.05–F_ (MPa)	n_0.05–F_ (-)	K_0.1–F_ (MPa)	n_0.1–F_ (-)	EA_TRA0.002–F_ (N.m)	EA_TRA0.05–F_ (N.m)	EA_TRA0.1–F_ (N.m)
HSLA 240	719	0.178	718	0.177	714	0.174	104.5	104.6	104.6
DP 600	1080	0.213	1073	0.209	1051	0.198	144.4	144.8	145.5
TRIP 400/700	1386	0.260	1460	0.286	1432	0.275	187.5	186.7	187.5

Note: *K_interaval_*—strength coefficient; *n_.interval_*—strain hardening exponent; *EA_TRAinterval_*—energy absorption evaluated by regression analysis; *0.002–F*—interval within 0.002 strain to fracture strain; *0.05–F*—interval within 0.05 strain to fracture strain; *0.1–F*—interval within 0.1 strain to fracture strain.

**Table 4:** Measured and calculated values of the deformation characteristics by the three-point bending test.

	Measured and Calculated Data						
	x_plmax_	EA_b.CTM_	EA_b.RA_	F_bmax_	CS_b.EA_	CS_b.RA_	CS_b.RA_/CS_b.EA_
Material	(mm)	(Nm) Acc. to Equation (9)	(Nm) Acc. to Equation (14)	(kN)	(kN/m) Acc. to Equation (13)	(kN/m)	(-)
HSLA 240	38.5	285.4	279.0	13.89	385.1	377.4	0.980
DP 600	35.5	374.4	308.2	17.76	594.2	489.1	0.823
TRIP 400/700	40.0	535.1	589.8	24.45	668.9	612.2	0.915

**Table 5:** The strain values measured by the 3D optical system Argus.

Strain Zone	Strain	Material		
		HSLA 240	DP 600	TRIP 400/700
Safe zone SP	Major φ_T1_ (-)	0.26 ± 0.02	0.15 ± 0.02	0.16 ± 0.02
	Minor φ_T2_ (-)	−0.12 ± 0.01	−0.07 ± 0.01	−0.08 ± 0.01
	Thickness φ_T3_ (-)	−0.14 ± 0.01	−0.08 ± 0.01	−0.08 ± 0.01
Warning zone WP	Major φ_T1_ (-)	0.33 ± 0.02	0.23 ± 0.01	0.21 ± 0.02
	Minor φ_T2_ (-)	−0.16 ± 0.01	−0.11 ± 0.01	−0.12 ± 0.01
	Thickness φ_T3_ (-)	−0.17 ± 0.01	−0.12 ± 0.01	−0.09 ± 0.01

**Table 6:** Deformation characteristics determined by simulation of the three-point bending test.

Material	Measured and Calculated Data						
	x_pl.s_	EA_s.b_	EA_s.b_/EA_b.CTM_	F_s.b_	CS_s.b_	CS_s.b_/CS_b.EA_	x_pl.s_/x_pl.b_
	(mm)	(Nm)	(-)	(kN)	(Nm)	(-)	(-)
HSLA 240_0.002–F_	40	222.61	0.78	12.37	295	0.766	1.04
HSLA 240_0.05–F_	40			12.39			
HSLA 240_0.1–F_	40			12.23			
DP 600_0.002–F_	47	340.70	0.91	17.62	369.8	0.622	1.32
DP 600_0.05–F_	45			17.64			1.30
DP 600_0.1–F_	46			17.08			1.27
TRIP 400/700_0.002–F_	52	470.89	0.88	24.63	509	0.761	1.30
TRIP 400/700_0.05–F_	57			26.37			1.43
TRIP 400/700_0.1–F_	56			25.98			1.40

**Table 7:** Comparison of values FLC0_WP_.

Data from	HSLA 240	DP 600	TRIP 400/700
Measured	0.22	0.205	0.2
Simulation	0.21	0.275	0.275
Acc.to [34,35,36]	0.27 [34]	0.18 [35]	0.18 [36]

## Data Availability

Data available on request due to restrictions of funder.
